# Insight in Superiority of the Hydrophobized Gentamycin in Terms of Antibiotics Delivery to Bone Tissue

**DOI:** 10.3390/ijms232012077

**Published:** 2022-10-11

**Authors:** Konrad Kwiecień, Iwona Pudełko, Karolina Knap, Katarzyna Reczyńska-Kolman, Małgorzata Krok-Borkowicz, Dorota Ochońska, Monika Brzychczy-Włoch, Elżbieta Pamuła

**Affiliations:** 1 Department of Biomaterials and Composites, Faculty of Materials Science and Ceramics, AGH University of Science and Technology, Al. Mickiewicza 30, 30-059 Kraków, Poland; 2 Department of Molecular Medical Microbiology, Chair of Microbiology, Faculty of Medicine, Jagiellonian University Medical College, 18 Czysta Street, 31-121 Kraków, Poland

**Keywords:** hydrogels, bone tissue, nanoparticles, PLGA, gentamycin, AOT, MRSA

## Abstract

Bone infections are a serious problem to cure, as systemic administration of antibiotics is not very effective due to poor bone vascularization. Therefore, many drug delivery systems are investigated to solve this problem. One of the potential solutions is the delivery of antibiotics from poly(L-actide-co-glycolide) (PLGA) nanoparticles suspended in the gellan gum injectable hydrogel. However, the loading capacity and release kinetics of the system based on hydrophilic drugs (e.g., gentamycin) and hydrophobic polymers (e.g., PLGA) may not always be satisfying. To solve this problem, we decided to use hydrophobized gentamycin obtained by ion-pairing with dioctyl sulfosuccinate sodium salt (AOT). Herein, we present a comparison of the PLGA nanoparticles loaded with hydrophobic or hydrophilic gentamycin and suspended in the hydrogel in terms of physicochemical properties, drug loading capacity, release profiles, cytocompatibility, and antibacterial properties. The results showed that hydrophobic gentamycin may be combined in different formulations with the hydrophilic one and is superior in terms of encapsulation efficiency, drug loading, release, and antibacterial efficacy with no negative effect on the NPs morphology or hydrogel features. However, the cytocompatibility of hydrophobic gentamycin might be lower, consequently more extensive study on its biological properties should be provided to evaluate a safe dose.

## 1. Introduction

Despite the advanced natural capability of bone tissue to regenerate, only simple damages may be regrown without surgical intervention. Although it is still unclear what the size of the defect is that would not be able to be repaired by natural mechanisms only, the estimated value of the so-called critical defect is estimated to be around 2.5 cm [1]. Some serious large bone defects may not only come from injuries but also from infections, tumor resections, trauma, etc. The use of different bone autografts or allografts remains a gold standard in such cases; however, it is not free from limitations and new approaches are required in this field, making bone tissue engineering of great interest in biomedical studies [2,3]. Moreover, the market for tissue engineering is constantly growing. In 2017, the sales of tissue engineering related products generated an estimated value of $9 billion in the U.S. only [4].

Surgical interventions may cause post-operative infections the risk of which is determined by many factors, e.g., age, biomechanical stability of the graft, or alcohol consumption [5,6,7,8]. Systemic administration of antibiotics is not effective due to limited bone vascularity and therefore low supply on the side of action [9,10]. To overcome this problem, local drug delivery is investigated. There are plenty of possible biomaterials that may be used for this approach, e.g., bone cement, drug-eluting scaffolds, hydrogels, and nano- or microparticles (NPs or MPs, respectively) to be suspended in hydrogel or immobilized on the surface of the scaffolds [11].

NPs (or MPs) as carriers of antibiotics are especially interesting. Typically, they are manufactured from biodegradable polymers either natural (e.g., chitosan, silk fibroin) or synthetic (e.g., poly(lactide-co-glycolide)(PLGA), poly(ε-caprolactone)(PCL)) loaded with, e.g., gentamycin (Gent) [12,13,14,15,16,17,18,19,20], vancomycin [21,22,23,24,25], or other antibiotics [26,27]. Among possible materials, PLGA is highly biocompatible with tailorable degradation kinetics by adjusting molecular weight and copolymer ratio. Moreover, it is approved by the U.S. Food and Drug Administration (FDA). Therefore, it seems to be a reasonable choice for the manufacture of the NPs for antibiotic delivery purposes and it has been widely investigated in bone tissue engineering to date [28,29].

Current studies on encapsulating Gent within PLGA NPs and MPs show a relatively high, considering the hydrophilic character of the drug and hydrophobic character of the polymer, encapsulation efficiency (EE) of typically up to around 50% which leads to satisfactory drug loading and required burst release to prevent postoperative infections. On the other hand, in most cases there is a rapid or at least significant decrease in drug in vitro release after around 3 weeks [14,16,17]. This could be too short for a seriously damaged bone to repair, and the implanted area may still be prone to infections. A possible solution may be found by changing the character of the drug from hydrophilic to hydrophobic by ion-pairing. This approach was, to the best of our knowledge, introduced in 1997 by Falk et al. [30]. The team from Colorado, U.S., provided a reaction in which they changed Gent sulfate (GentS) into gentamycin bis-2-ethylhexyl sulfosuccinate (GentAOT). After encapsulation of the modified Gent in poly(lactic acid) (PLA), they obtained a formulation with a sustainable release for 70 following days. In 2011, Imbuluzqueta et al. [31] used the same approach for GentS ion pairing and encapsulated the product (GentAOT) within PLGA NPs, obtaining almost 100% of EE and continuous drug release also for the following 70 days and also confirmed its efficacy in vivo 2 years later [32]. In 2015, Boo et al. [33] produced GentAOT with that technique, confirmed its bactericidal properties against *Staphylococcus aureus* and *Staphylococcus epidermidis*, and encapsulated it in poly(trimethylene carbonate) matrices for orthopedic infections. Recently, in 2020 Rotman et al. [34,35] published two papers in which they encapsulated GentAOT in MPs from different biopolymers (poly(D,L-lactic acid) (PDLLA), PCL [34], and PCL modified with poly(aspartic acid) [35]) dedicated for the treatment of bone infections. The drug release kinetics published in the works were faster. In two weeks, all drug was released from poly(aspartic acid) PCL MPs, while in pure regular PCL MPs released up to 50% or 70%—depending on the fabrication method. PDLLA MPs released GentAOT in the slowest manner. Two-week incubation led to release of up to 30% or 50% also depending on the fabrication method. These results show that GentAOT may be used for prolonged antibiotic release in the drug delivery systems for bone tissue. It was also shown that the choice of the material and the fabrication has a strong impact on the release kinetics. Although GentAOT has been appearing in the literature for 25 years now, there are not many manuscripts on it, creating a niche for future studies. Especially, AOT has recently been used to change the chemical character of other drugs—another antibiotic: tobramycin [36] and chemotherapeutic: chlorambucil prodrug [37].

We hypothesize that using the combination of hydrophilic and hydrophobic gentamycin will allow to provide a more efficient composite drug delivery system consisting of hydrophilic hydrogel and hydrophobic NPs. The high affinity of the drugs to their carriers will result in high drug loading capacity. The aqueous environment of human body, on the other hand, will cause a fast burst release to form the hydrophilic carrier continued by sustained and prolonged release due to slow hydrolytic degradation of PLGA NPs. Therefore, the aim of this study was to obtain a drug delivery system suitable for particular clinical needs of bone tissue infections. In order to provide it, GentAOT was obtained and encapsulated in PLGA NPs using solid-in-oil-in-water (S/O/W) emulsification. Then, the NPs were investigated for EE and drug loading (DL), size distribution (DLS), and morphology (SEM). After that, the NPs were suspended in gellan gum hydrogel as previously described [20,25], loaded with GentS for initial burst release, and GentAOT-loaded NPs for prolonged sustained release. The biocompatibility of the system was evaluated with the osteoblast-like MC3T3 cell line and the bactericidal properties with methicillin-resistant *Staphylococcus aureus* (MRSA) were evaluated. We hypothesize that using the combination of hydrophilic and hydrophobic gentamycin will provide more sufficient antibacterial effect in terms of drug release and efficacy.

## 2. Results

### 2.1. Synthesis and Bactericidal Properties of GentAOT

**Figure 1:**
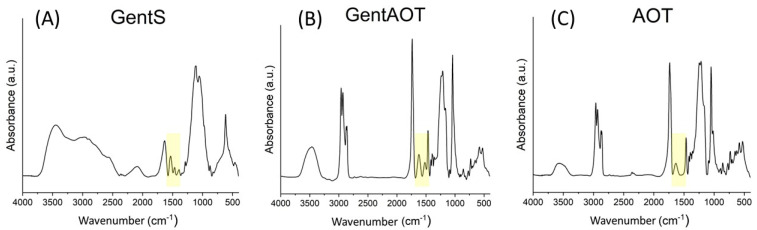
FTIR spectra of (**A**)—GentS, (**B**)—GentAOT with red arrow showing the additional band at 1620 cm^−1^, (**C**)—AOT.

GentS was successfully transferred to GentAOT, as confirmed by Fourier-transformed infrared spectroscopy (FTIR) (Figure 1). The FTIR spectrum of GentAOT is very similar to pure AOT. AOT—unlike GentS—does not have any amine groups in its structure, which can be assigned to, e.g., the absorption band with maximum at 1620 cm^−1^. This and a few other bands marked by a yellow color in Figure 1 in both GentS and GentAOT spectra, and not present in AOT spectra, consists of a sufficient proof of antibiotic modification according to Boo et al. [33] who got similar results.

**Figure 2:**
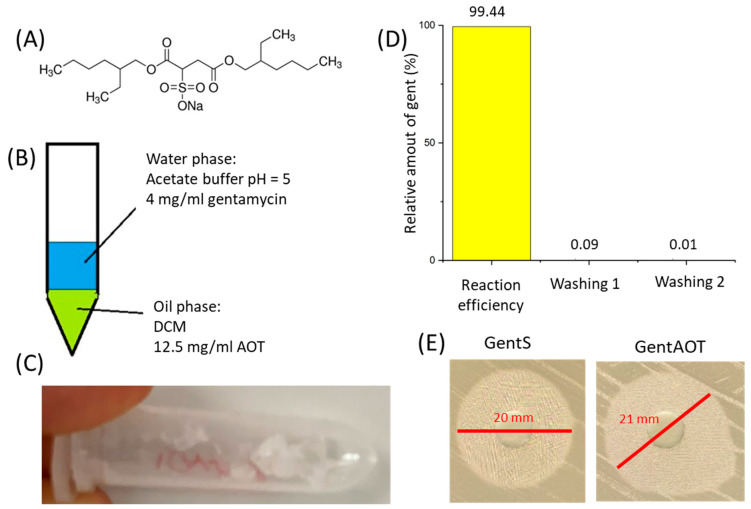
(**A**)—Chemical formula for AOT, (**B**)—schematic setup for manufacturing GentAOT, (**C**)—the product of ion-pairing right after DCM evaporation, (**D**)—Efficiency of the ion-pairing and losses during two washings, (**E**)—antibacterial efficacy of the product compared to the substrate.

GentAOT was a sticky jelly-like form (Figure 2C). The efficiency of the reaction was assessed by the OPA assay and was 99.44% ± 0.04%, as only 0.56% of the initial GentS was evaluated to be left in the water phase after separation. Additionally, the losses during both washing were marginal—0.09% ± 0.00% and 0.01% ± 0.00% of initial GentS was transferred to the supernatants during the first and the second washing, respectively (Figure 2D).

The bactericidal properties of GentAOT in relation to *S. aureus* (NCTC 12973) were already confirmed by Boo et al. [33] who concluded that the efficiency of GentAOT in inhibiting the growth of this strain of bacteria does not change during the manufacturing process. In our study, the efficiency against MRSA (ATCC BAA 1681) was slightly higher, as the inhibition zone of 10 µg of GentAOT was 21 mm, whilst for the substrate GentS it was 20 mm (Figure 2E). The result is especially promising, minding the fact that the molecular mass of GentAOT is around twice as high as that of GentS, therefore in the same weight of GentAOT there is significantly less gentamycin molecules. In spite of that fact, GentAOT inhibits MRSA growth as efficiently as GentS or even better.

### 2.2. NPs Morphology and Size Distribution

Comparison between NPs obtained under the same conditions with GentS and GentAOT has been provided to assess the differences caused by the antibiotic complex on various physicochemical and biological properties. For this purpose, all of the manufactured batches of NPs have been investigated by the DLS technique for their size distribution and zeta potential. To visualize the particles and assess their morphology, SEM observations have been provided.

**Figure 3:**
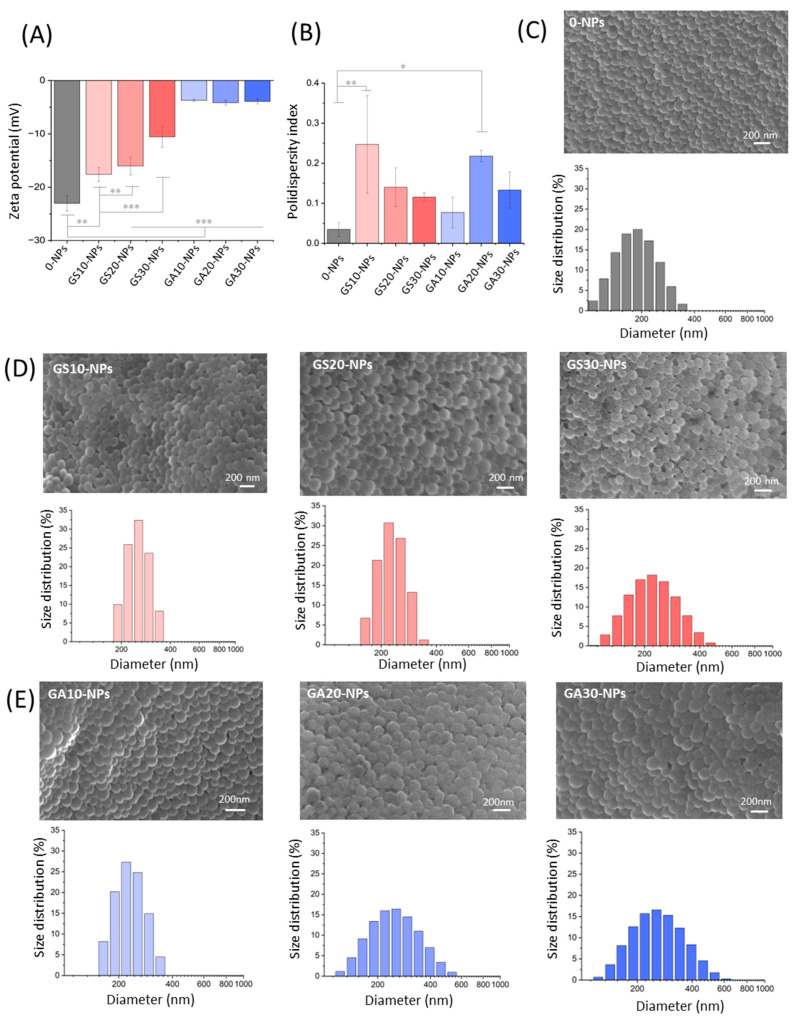
DLS measurements and SEM observations of obtained NPs. (**A**)—zeta potential of all NPs, (**B**)—polydispersity indexes of obtained NPs, (**C**)—morphology and size distribution of empty NPs, (**D**)—morphology and size distribution of GentS-loaded NPs, and (**E**)—morphology and size distribution of GentAOT-loaded NPs; * *p* < 0.05, ** *p* < 0.01, *** *p* < 0.001.

Scanning electron microscopy showed that all batches obtained were spherical in shape and similar to each other. The surface appeared to be smooth; however, the high contrast could not have been acquired because of the material swelling. The pictures were, therefore, taken with a low voltage of 5 mV, resulting in a not very high resolution (Figure 3C–E).

It appears that the most negatively charged particles are the empty ones. The addition of any type of the drug reduces the zeta potential, but in no case were the NPs neutral or positively charged. The presence of GentS leads to a gradual reduction of the negative surface charge from −23.0 ± 1.4 mV for the empty NPs to −10.6 ± 2.0 mV for the GS30-NPs, respectively. In contrast, GentAOT drastically reduced the charge to a value of around −4 mV, irrespective of the concentration used. It seems that the presence of AOT has a predominant influence on the surface charge, making a significant difference (Figure 3A). On the other hand, the fact that one can expect very similar results despite the different drug additions, makes it easier to forecast the value of formulation’s zeta potential.

The encapsulated drugs also influenced the NPs size distribution. The batch of 0-NPs appeared to be clearly the smallest with the average size ± SD equal to 179.4 ± 0.9 nm. All drug-loaded NPs ranged from 211.1 ± 1.9 nm to 262.4 ± 8.2 nm (Figure 3C–E). However, neither the choice of the drug nor its concentrations appeared to make a significant difference. Because no trend has been observed, we concluded that the variability of the NPs comes from the limited repeatability of the manufacturing method, because most of the steps were done manually. This interpretation is supported by the polydispersity indexes (PdI) of the batches that varied for 0-NPs and drug-loaded NPs. However, there is no trend in the difference. Statistically different PdIs, according to 0-NPs, are GS10-NPs and GA20-NPs (Figure 3B) showing no visible signs of dependence between PdI and the choice or amount of the drug used.

### 2.3. Encapsulation Efficiency and Drug Loading

The supernatants of all batches have been investigated to assess the EE and DL of the GentAOT and GentS entrapment. For this purpose, the OPA fluorescent assay has been adjusted to the solubility of the drugs. In Table 1 the calculated values of EE and DL (average ± SD) have been set together.

Although all of the manufacturing parameters are the same for both GentAOT- and GentS-loaded NPs, the resulting DL has always been several times higher in the case of the use of the hydrophobic complex. The EE of the GentAOT was almost complete and more repeatable than that of GentS which varied in no reasonable manner. Theoretically, the EE should gradually decrease at higher concentrations, but at some point, there should be an increase in DL so that the most effective concentration can be optimized [38]. In our case, the lowest concentration of GentS led to the lowest EE raising a question about the repeatability of the method. For GentAOT, the EE is not only of a nearly theoretical value but also seems to be much more repeatable, as all the results are very similar. Furthermore, no decrease in EE while increasing the drug concentration suggests that the final DL may increase even more at higher initial concentrations, giving a variety of possibilities to adjust the DL almost freely.

### 2.4. Hydrogel Characteristics

**Figure 4:**
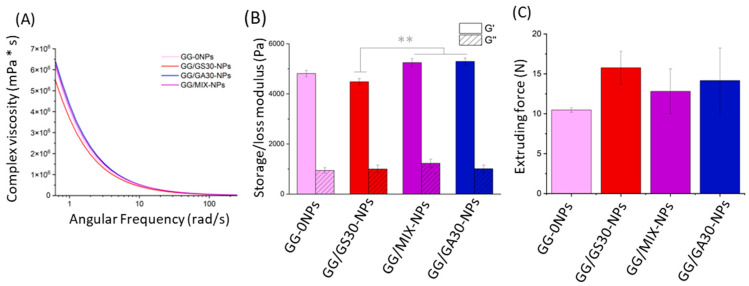
Characteristics of hydrogels. (**A**)—Complex viscosity as a function of angular frequency, representative samples, (**B**)—mean storage (G’) and loss (G”) modulus with SEM, (**C**)—mean maximal extruding with SEM. Statistical significance was measured by one-way-ANOVA with Tukey test; ** *p* < 0.01.

In order to evaluate the influence of GentAOT-loaded NPs on hydrogels features, complex viscosity and injectability were measured. As shown in Figure 4A, viscosity of the hydrogels was decreasing with the increasing shear rate. All the hydrogels behaved in a similar manner. The initial viscosity of samples with GentAOT (GG/GA30-NPs and GG/MIX-NPs) was slightly higher.

The lowest value of storage modulus (G’) occurs for GG/GS30-NPs, whereas the highest for the sample with GentAOT-loaded NPs (Figure 4B). Furthermore, sample containing only GentS-loaded NPs differs from GG/MIX-NPs and GG/GA30-NPs, significantly, while there is no relevant difference in the case of GG-0NPs. In the case of loss modulus (G”), there is no significant difference regardless of the type of NPs present in hydrogels.

The maximal extruding forces for hydrogels varied from 10 to 18 N, showing they are easily injectable. Despite the various types of NPs there were no significant differences in terms of maximal forces (*n* = 3). The presence of both GentS and GentAOT in NPs seemed to lead to a slight increase of the maximal extruding force; however, the differences were not statistically significant (α = 0.05).

Interestingly, while the results showed that the presence of GentAOT in the formulations leads to higher viscosity in low angular rates, it does not significantly affect the extruding force in comparison with GentS-loaded NPs (Figure 4C). It suggests that modification of GentS has no negative impact on injectability of hydrogels, but this speculation should be further investigated.

### 2.5. In Vitro Drug Release

**Figure 5:**
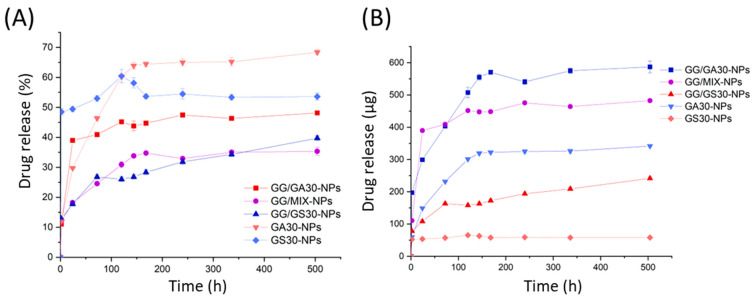
The release kinetics of different NPs in either PBS suspensions or GentS-loaded GG hydrogels suspensions. (**A**)—relative drug released from the formulations, (**B**)—amount of drug released from the formulations.

In order to study the drug release, different NPs-based systems were placed in dialysis bags and kept in PBS while constantly stirred. At numerous time points, the concentration of gentamycin within the solution was evaluated. The amount of drugs released from all the five hydrogel formulations (GS30-NPs, GA30-NPs, GG/GS30-NPs, GG/MIX-NPs, GG/GA30-NPs) was expressed as a relative (Figure 5A) and absolute (Figure 5B) value.

All formulations released gentamycin in a similar manner. The initial burst release is clearly visible for all the systems. However, for the samples with GentAOT it is prolonged —GS30-NPs released nearly 50% of the drug within the first 2 h, whilst the GA30-NPs provided an enhanced release for the first 5 days of incubation. The hydrogel samples also liberated gentamycin in a similar manner. The higher relative release in the case of GG/GA30-NPs might be a result of small differences in the NPs to free drug ratio, as in that case weighed amount of free GentS was slightly higher. The drug leaves hydrogels relatively slower, as the NPs have no direct contact with PBS. That is why the pick visible in the case of GS30-NPs suspension is not visible in the case of GG/GS30-NPs.

The biggest differences are in the absolute amount of drug delivered to the PBS. GS30-NPs after their initial burst do not seem to really deliver any significant amount of drug later on. GA30-NPs, on the other hand, deliver a reasonable dose of the drug for 5, up to 6, days. From this moment on, the continuous release of small doses was noticed. For hydrogels, the burst release time was slightly dependent on the type of NPs used. The process decelerated after 3 days in the case of GG/30GS-NPs, 5 days for GG/MIX-NPs, and 6 days for GG/GA30-NPs, respectively. After that time, all the formulations delivered the drug continuously in similar small portions. The differences in the absolute values proved that the release from hydrogels, even shortly after the beginning of the incubation, is simultaneously from the GG and NPs because all the hydrogels had similar loading of free GentS.

### 2.6. Cytocompatibility

**Figure 6:**
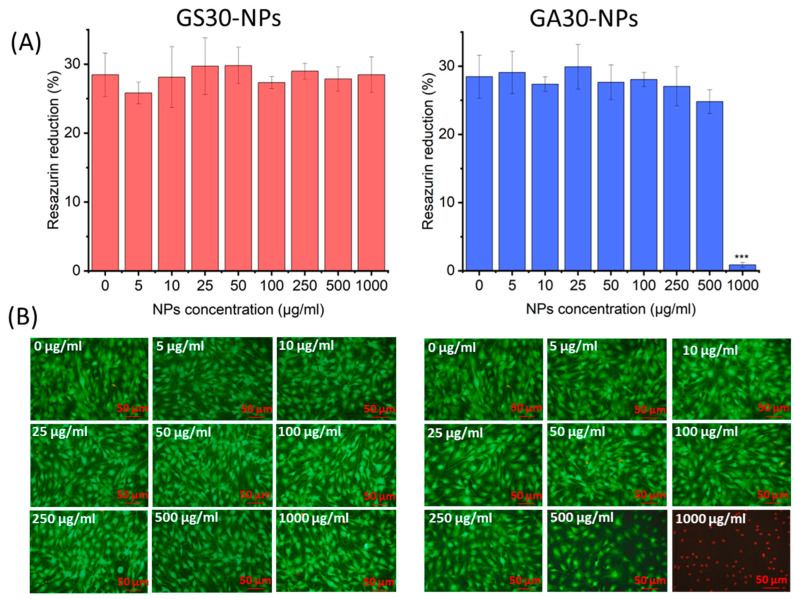
The cytocompatibility test of GS30-NPs and GA30-NPs. (**A**)—AlamarBlue results Average ± SD (*n* = 3), (**B**)—live/dead fluorescent staining—merged pictures of living (green) and dead (red) cells. Statistical significance was measured by one-way-ANOVA with Tukey test, *** *p* < 0.001.

To evaluate the difference in cytocompatibility between the GentAOT- and GentS-loaded NPs, the metabolic test AlamarBlue and live/dead fluorescent staining were performed. GS30-NPs showed no cytotoxic effect even in the highest concentration of 1000 µg/mL. On the contrary, GA30-NPs were safe for MC3T3 cells up to 500 µg/mL. At a concentration of 1000 µg/mL, almost no living cells were found after 3 days of incubation (Figure 6A). Live/dead staining was compatible with the AlamarBlue assay (Figure 6B). In all cases, apart from GA30-NPs at 1000 µg/mL, the cells looked similar. They were densely distributed throughout the wells, flattened with dead cells appearing very rarely. Only in the wells with the cells incubated with GA30-NPs at 1000 µg/mL, finding living cells was hardly possible, as almost all of them were dead.

Both samples showed relatively high cytocompatibility. However, GA30-NPs appeared to be more toxic than GS30-NPs in the case where the cytocompatibility limit was not reached in the experiment. The question is whether the difference comes from the presence of AOT or the much higher concentration of gentamycin in the solution. The assessed DLs were much higher in the case of GentAOT-loaded NPs. Based on DL and release studies it could be estimated that the concentration of gentamycin in the wells with 1000 µg/mL GS30-NPs after 72 h could be around 25 µg/mL whilst in the wells with 1000 µg/mL GA30-NPs around 112 µg/mL, making a significant difference. On the one hand, Boo et al. [33] suggested that the increased bactericidal properties of GentAOT could have increased the potential to cross the cell membrane and showed that it is more toxic to hTERT fibroblast cells. On the other hand, there are different studies focusing on the cytotoxicity of pure gentamycin toward various cell lines. For example, gentamycin at a concentration of 100 µg/mL did not affect the viability of human mesenchymal stem cells (hMSC) but decreased their proliferation [38]. The hydrophobicity of the GentAOT did not affect the burst release from NPs because a significant amount of drug is probably accumulated on the surface. Much different drug loadings lead to completely different release profiles so that even if the GentAOT-loaded NPs are more toxic, significantly less of them could be used to obtain the same effect as in GentS-loaded NPs. The safe concentration limit of 500 µg/mL is high anyway. Therefore, GentAOT-loaded NPs are still promising, but more detailed study on the safe dosage of GentAOT is needed.

### 2.7. Bactericidal Properties Evaluation

**Figure 7:**
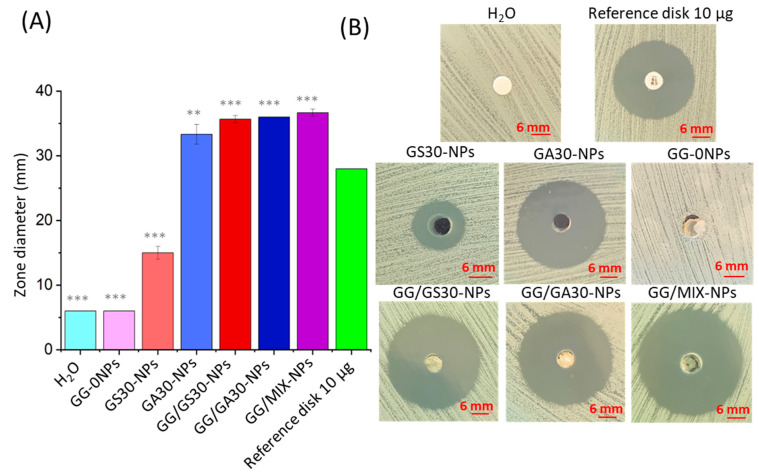
Kirby-Bauer (agar diffusion test) results. (**A**)—mean values of inhibition zone widths (n = 3, ± SD) with a statistical difference with the standard 10 µg gentamycin paper disc, (**B**)—the representative pictures of inhibition zones. Statistical significance was measured by one-way-ANOVA with Tukey test; ** *p* < 0.01, *** *p* < 0.001.

The antibacterial properties of the systems were evaluated using Kirby-Bauer (agar diffusion test) with MRSA. In the 6 mm holes cut out with a sterile pipette tip in the Mueller-Hinton agar, the samples in the forms of NPs suspensions in PBS or GentS-loaded hydrogels were placed. All samples with encapsulated gentamycin of either form showed some inhibition zones in the growth of MRSA (Figure 7). In the case of NPs suspensions, there is a clear difference between GA30-NPs and GS30-NPs that showed 33 mm and 16 mm of the inhibition zones, respectively. The result of a more than twice as wide inhibition zone for GentAOT shows its much higher DL capacity with no harm to release kinetics. The hydrophobic character of GentAOT did not suppress the initial burst capability of NPs that is necessary in the infected area. Moreover, according to the European Committee on Antimicrobial Susceptibility Testing (EUCAST) [38], the minimal inhibition zone to accept a factor sufficient against S. aureus is 18 mm so the GS30-NPs with an average result of 15 mm do not count as sufficient. All the hydrogel samples showed similar inhibition zones (in the range of 35–38 mm), as the first burst is practically related only to GentS dissolved in the GG hydrogel. At the beginning of treatment, there would be no difference between GG/GS30-NPs and GG/GA30-NPs. However, in the further stages GentS-loaded NPs might not be enough, even though they may show a sustained release [17,20], as they are not sufficient according to the norm. Even in the composite system, the NPs themselves should be able to treat the infection independently, as in vivo conditions would cause a higher elution rate rather than in vitro release studies.

## 3. Discussion

The aim of the study was to obtain a hydrophobic complex of gentamycin—GentAOT—encapsulate it in PLGA NPs and compare them with analogical NPs loaded with nonmodified gentamycin in the terms of their morphology, drug loading capacities, release kinetics, cytocompatibility, and antibacterial properties. In the literature, there are several studies dedicated to the different formulations for the delivery of GentAOT. To the best of our knowledge, none of them focus on a direct comparison between the NPs obtained with GentAOT and GentS as potential drug delivery systems to bone tissue.

GentAOT was obtained successfully, and it was confirmed that it is not losing its efficiency against MRSA. So far, the efficiency of the reaction was evaluated by infrared spectroscopy (FTIR) [33] and it was successfully repeated by us. However, the AOT spectra is dominating the spectra of GentS thus there are not many differences between the spectra of AOT and GentAOT. The indicating factor here is the only one absorption band between at 1620 cm^−1^ which comes from the first-order amine group of gentamycin [33]. This observation somehow proves the presence of gentamycin in the product of ion pairing but gives no quantitative picture of the yield. In this study, we used the OPA assay to investigate the water phase, in which GentS was dissolved prior to the reaction, after the process was completed. We confirmed that there is, in fact, a very small amount of GentS that do not undergo the ion pairing (around 0.5%). We also proved that the product can be safely washed without significant loss of the gentamycin content from GentAOT.

The use of GentAOT showed a very high EE of almost 100% which is similar to the result obtained by Imbuluzqueta et al. [31,32], who also obtained impressively high DL values of up to 60%. It supports the assumption that PLGA can mix with GentAOT freely, giving an opportunity to obtain almost any desired DL. Such values are superior to GentS-loaded NPs. In our study, we were able to receive NPs with DL not exceeding several percent. Moreover, the repeatability between the batches seems to be low due to the low drug to polymer affinity. Posadowska et al. [17] worked with GentS-loaded NPs and also obtained a wide range of EE and DL values of 2.7% ± 1.8% to 52.4% ± 0.5% and 0.06% ± 0.05% to 10.28% ± 0.04%, respectively. Jiang et al. [39] used GentS-loaded NPs of only 2.2% DL in their studies. Abdelghany et al. [40] also created GentS-loaded NPs that did not reach much above 2% of the DL. Dhal and Mishra [41], on the other hand, made an impressively wide optimization study and obtained EE values from 0.00% to 90.10%. It seems that the final DL is highly dependent on even minor changes in manufacturing parameters not being very convenient when it comes to provide assured, attested formulations for clinical use. GentAOT, in contrast, would give the same result even if some manufacturing parameters differ [31], providing predictable results every time.

The use of GentAOT-loaded NPs did not influence negatively the features of hydrogel itself. The tests of viscosity and injectability did not show significant changes in the samples in which GentAOT was applied (i.e., GG/MIX-NPs and GG/GA30-NPs). Therefore, obtained differences should not affect the practical use of the formulations, as all the samples had similar dependence between complex viscosity and angular frequency in the rheological test and they were injectable while applying relatively low forces. In addition, obtained results are lower than maximal extrusion force of the system for surgical manipulation (20 N) [20]. Moreover, modification of GentS showed no relevant differences in terms of either storage or loss modules. On the other hand, the changing in extruding force during injecting suggests that NPs were probably not perfectly homogenously distributed in the hydrogel. Therefore, it could be useful to improve the manufacturing method, though we believe it is rather the technological challenge than any influence of chosen drug.

The release studies showed that GentAOT-based formulations are able to deliver the higher amount of drugs in a more sustainable manner. The initial burst could be prolonged twice by using GA30-NPs instead of GS30-NPs in the GG hydrogel. This could be more effective in the treatment of a serious infection that may require a higher concentration of antibiotics for a longer period. Posadowska et al. [17] obtained better release profile for GentS-loaded NPs that continuously released the drug for 3 weeks before the process decelerated. In this study, they used double emulsification to manufacture the NPs that could have led to more bulk encapsulation, while our NPs may have more of the GentS on the surface. On the other hand, with the differences between various NP formulations in mind, the question of repeatability might be raised. They also showed that the GentS based system similar to GG/GS30-NPs can release the drug in a sustainable manner for more than 80 days after the initial burst [20]. According to that result, the biggest advantage of the use of GentAOT in that case was obtaining the prolonged burst release that should provide enough time to cure the acute infection, as the doses themselves could be adjusted easily unlike the release kinetics.

These advantages do not have a significant influence on the NPs morphology. The diameter sizes of the NPs were similarly influenced by both GentAOT and GentS. The SEM observations also did not show a visible effect of the choice of the drug. Only the values of the zeta potential were significantly different. The highest negative potential was observed for the 0-NPs. While the initial amount of GentS was increased, a gradual reduction of the potential was observed. However, there was no statistically significant difference between the GentAOT concentration, making this kind of NPs much more predicable in this case. Such an observation is consistent with the study by Imbuluzqueta et al. [31] on GentAOT-loaded PLGA NPs.

With the superior loading capacity of GentAOT comes also a much higher antibacterial efficacy. GA30-NPs could inhibit the MRSA growth much better than the GS30-NPs which did not fulfil the norm conditions [38]. In the literature, the antibacterial efficacy has already been confirmed in contact with different bacterial strains, e.g., *S. aureus S. epidermidis* [33], *Escherichia coli* [35], and *Brucella* [32]. No studies suggested that the efficacy of GentAOT would decrease compared to GentS. For the fresh hydrogel samples, the obtained results were very similar, irrespective of the chosen NPs. Because the antibacterial activity of NPs alone differed much, it indicates that all the initial inhibitory effect was the result of GentS dissolved within the gellan-gum. It can be assumed that it is an analogy to the studies of Posadowska et al. with either gentamycin [20] or vancomycin [25], according to which the hydrogel samples were prepared. Hydrophilic drugs are quickly released from the hydrogels assigning the importance of the role of the NPs in the later stages of sustained release. For this purpose, the chosen NPs should be characterized with high DL and prolonged release. Release studies showed that the burst release appeared for GentAOT-loaded NPs too, because probably a significant part of the drug was adsorbed to the surface. Dispersing in the hydrogel allows the process to decelerate, and slowly degrading NPs should provide a significant amount of drug in the subsequent stages of the therapy.

The only issue related to the use of GentAOT is not fully evaluated biocompatibility. Boo et al. [33] noticed that pure GentAOT is more toxic than GentS in contact with hTERT fibroblast cells. The results showed that the concentration of 11 µM, which corresponds to around 20 µg/mL of GentAOT decreased cell viability to around 50%. Nonmodified gentamycin tested with other different types of cells is usually higher (50 µg/mL of gentamycin safe for rabbit corneal epithelial cells [42], hMSC cells—viability not affected in 200 µg/mL, proliferation not affected in 50 µg/mL gentamycin [43]). On the other hand, Kumar et al. [44] claimed that 1 mM gentamycin is toxic to Vero kidney cells and based their study on protecting the cells from this damage. Studies by Rotman et al. [34,35] gave very promising results, but did not focus much on the toxicity aspect. Our study showed that the GentAOT-loaded NPs are superior to the GentS-loaded ones in many aspects. They showed higher cytotoxicity than GS30-NPs, though. However, Imbuluzqueta et al. [32] tested GentAOT in vivo obtaining no signs of toxicity.

All of this leads to the commonly known fact that each substance is a poison at a high enough concentration. As it seems that GentAOT is in fact more toxic than GentS, the main question is whether the doses to be applied would be below that limit and the therapeutic effect may be obtained without causing damage to healthy tissues. To answer this question, more detailed studies of this hydrophobic complex are required. However, GentAOT should not be overlooked by scientists because of its impressively high loading capacity connected with repeatability and sufficient release kinetics.

## 4. Materials and Methods

### 4.1. Materials

Poly(lactide-co-glycolide) (PLGA, La:Ga ratio 85:15) was synthesized in Polish Academy of Sciences, Zabrze, Poland. Gentamycin sulfate, dioctyl sulfosuccinate sodium salt (AOT), gellan gum (GG, GelzanTM, MW 200–300 kDa), poly(vinyl alcohol) (PVA, Mowiol 4–88), o-phtaldialdehyde, 2-merkaptoethanol, calcein-AM, propidium iodide, resazurin, potassium bromide (KBr), and calcium chloride (CaCL_2_) came from Sigma-Aldrich (Steinheim, Germany). Dichloromethane (DCM) and methanol were provided by Chemland (Stargard, Poland). Acetic acid and sodium acetate for buffer solution came from POCH (Gliwice, Poland). Modified Eagle Medium (MEM) used for the cell culture was upplemented with 10% fetal bovine serum (FBS) and 1% of the mixture of penicillin and streptomycin (all chemicals from PAN Biotech, Aidenbach, Germany). Phosphate-buffered saline concentrate (PBS buffer) was provided by VWR Life Science (Radnor, PA, USA).

The osteoblast-like cell line MC3T3-E1 (ATCC CRL-2593). The bacteria were from the reference strain of methicillin-resistant *Staphylococcus aureus* (MRSA, ATCC BAA-1681) and were cultured in trypticasein soy broth (TSB, BioMaxima S.A., Lublin, Poland).

The reference strain used in the study was methicillin-resistant *Staphylococcus aureus* ATCC^®^ BAA-1681™ (MRSA, American Type Culture Collection, Manassas, VA, USA).

### 4.2. Synthesis of GentAOT

GentAOT has been obtained according to the recipe published earlier by Boo et al. [33]. Briefly, DCM solution of AOT (Figure 1A) (1.25% *w*/*v*) and water solution of GentS (0.40% *w*/*v*) at pH = 5, adjusted by acetate buffer solution, were mixed in equal volumes (Figure 1B). The mixture was stirred vigorously for 3 h and left until both phases separated completely. Then, the water phase was collected and saved for yield evaluation. The oil phase was left for DCM evaporation. The product of the reaction was collected, weighed, and washed 2 times in ultra-pure water (UHQ-water) (Direct-Q3UV, Merck Millipore, Burlington, MA, USA) by adding 1 mL of UHQ-water, shaking at Vortex for 10 min, and centrifugation—the supernatants after washing were also collected to evaluate the potential losses. The GentAOT obtained was lyophilized (Alpha 1–2LD plus DONSERV) and stored at −20 °C.

The yield of the reaction, as well as the losses in after-washing supernatants, were evaluated by the orto-pthaldialdehyde (OPA) assay. For this purpose, 30 mg of OPA was dissolved in 0.5 mL of methanol and 0.1 mL of mercaptoethanol and the solution was then added to 50 mL of boranic buffer (pH = 10.4). Such freshly obtained reagent was mixed with the samples in volumetric ratio 1:1 and incubated in the darkness for 10 min. After that the fluorescence was measured (FLUOstar Omega, BMG Labtech, Ortenberg, Germany) at the excitation wavelength in range of 340–310 nm and emission wavelength 460 nm.

The phenomenon of GentS modification with AOT was proved by Fourier transform infrared spectroscopy (FTIR) using (Bruker, Tensor 27. For this purpose, GentS, AOT, and GentAOT was dried overnight in a vacuum drier (SPU-200) prior to the experiment. Then, a ca. 2 mg of each sample was homogenized with 200 mg of potassium bromide (KBr) using a mortar. The resulting powder was pressed into a tablet and the IR absorbance was measured in transmutation mode at the wavenumbers of 4000–400 cm^−1^ (64 scans, resolution 4 cm^−1^). The data was processed by the OPUS software.

The antibacterial efficacy of GentAOT against methicillin-resistant *Staphyloccocus aureus*—MRSA (ATCC BAA 1681) was assessed using the Kirby-Bauer test. DCM solution of GentAOT was introduced to the sterile fresh standard disc (10 µg in 10 mL of DCM, 6 mm in diameter, Oxoid, Hampshire, UK). A standard disc with substrate GentS was prepared analogically in PBS solution (10 µg in 10 mL of PBS), and the efficacy was compared with the standard GentS disc (10 µg dose). The test was performed as described in sub Section 4.11 Bactericidal properties evaluation.

### 4.3. Synthesis of Polymeric Nanoparticles

Polymer NPs were received using the S/O/W emulsification with solvent evaporation method. For this purpose, the 2% solution of PLGA in DCM and the 2% solution of PVA in water were prepared. In our emulsion, the PVA solution acted as a surfactant. The GentS and the GentAOT were used as active substances that were encapsulated. To do so, the different amounts of either GentS or GentAOT (10, 20, or 30 mg of drug per 100 mg of PLGA) were emulsified in 3 mL of the PLGA solution in DCM. Homogenization was carried out using ultrasonic mixing for 3 min with an amplitude of 40% (Sonics, Vibra Cell VCX130, Newtown, CT, USA). Subsequently, the prepared emulsion was added to 20 mL of PVA solution and mixed with ultrasounds under the same conditions as for the first emulsion, followed by mixing on the magnetic stirrer (MS-52M, JeioTech, Daejeon, South Korea) for 24 h at a speed of 1000 rpm at room temperature. The emulsions obtained that way were then centrifuged at a speed of 15,000 rpm for 20 min at 4 °C (MPW-351R, MPW Med. instruments, Poland). Afterward, the supernatant was removed from the tubes, the UHQ-water was added, and another centrifugation under the same conditions was performed. To remove the PVA, the centrifugation with water was repeated four times. After the last one, the obtained NPs were frozen at −80 °C, left for 24 h, and then freeze-dried (Christ Alpha 1–2 LDplus, Osterode am Harz, Germany,). In Table 2, there are acronyms for NPs used throughout the article according to the type and amount of drug used to manufacture them.

### 4.4. Nanoparticle-Loaded Hydrogel Formulation

To receive hydrogel, gellan-gum (Gelzan^TM^, GG) was used. First, GG was dissolved in MilliQ-water, which was heated to 90 °C, to form a solution of 1.4% *w*/*v*. The mixture was left to decrease the temperature to 50 °C. A 0.3% *w*/*v* of CaCl_2_, 1% *w*/*v* suspension of NPs (GA30-NPs or GS30-NPs or a mix of both types), and 1 mg/mL of free antibiotic were weighed, added to the dissolved GG and mixed thoroughly. The amount of free antibiotic added was equal to the amount of GentAOT encapsulated in GA30-NPs. Table 3 shows the acronyms for the hydrogel samples used throughout the paper to refer to them.

### 4.5. Hydrogel Characterization

The influence of using GentAOT or GentS was also investigated in terms of hydrogel properties. Two features have been tested—injectability and viscosity. For these tests, GG/GA30-NPs, GG/GS-30NPs, GG/MIX-NPs, and GG/0-NPs had been chosen. The injectability was tested by placing the hydrogel samples into the 2 mL syringes equipped with standard needles 18 G. The syringes were placed vertically in the testing machine (Zwick 1435). A total of 1 mL of each hydrogel was extruded by compressive force with constant crosshead rate of 50 mm/min. Extruding force was measured continuously during the experiment. Then, the results were compared in terms of the maximal extruding force and average extruding forces were statistically analyzed for significant differences.

Rheological properties of unloaded hydrogels and hydrogels GA30-NPs, GS30-NPs, and 0-NPs were determined using a rheometer (MCR, Anton Paar, Graz, Austria) with temperature control (all the measurements were done at 20 °C), and a stainless-steel parallel plates (diameter: 20 mm) geometry. A frequency sweep test was performed using a 1000 µm gap, with a 0.75% oscillation strain and 0.1–100 Hz range. Each sample was tested twice. The average storage modulus (G′) and loss modulus (G′′) were calculated for the samples as well as the complex viscosity.

### 4.6. Encapsulation Efficiency and Drug Loading

To define the amount of antibiotics encapsulated in NPs, the orto-phtaldialdehyde (OPA) assay was performed. For GentS and GentAOT, the supernatants obtained after the first centrifugation during the preparation of NPs were tested. Briefly, the supernatants were mixed with the OPA reagent and fluorescently tested (FLUOstar Omega, BMG Labtech, Ortenberg, Germany). To do so, the OPA reagent for GentS was prepared according to the formula: 30 mg of o-phtaldialdehyde was mixed with 0.5 mL of methanol and 0.1 mL of 2-mercaptoethanol. After o-phtaldialdehyde was dissolved, 50 mL of borate buffer was added and thoroughly mixed. For GentAOT, as it is not soluble in water, 30 mg of o-phtaldialdehyde was dissolved in 50 mL of methanol. Then, 0.1 mL of 2-mercaptoethanol was added and thoroughly mixed.

Subsequently, 50 µL supernatant was mixed with 50 µL of one of the OPA reagents and incubated for 10 min. After that, the calibration curve was prepared, and the fluorescence of the supernatants was measured. The encapsulation efficiency (EE) and drug loading (DL) were calculated according to the following Equations (1) and (2), respectively. The fluorescence has been measured 3 times for each sample.

(1) $$\mathrm{EE}=\frac{mass\;of\;antibiotic\;in\;the\;NPs}{initial\;mass\;of\;antibiotic}*100\%\;$$

(2) $$\mathrm{DL}=\frac{mass\;of\;antibiotic\;in\;the\;NPs}{mass\;of\;NPs}*100\%$$

### 4.7. Size Distribution and Zeta Potential

The obtained NPs were tested for size using the dynamic light scattering DLS method. To do so, we suspended the NPs (each type separately) in MilliQ-water and subjected them to ultrasound for 10 min to shatter the agglomerates. Subsequently, suspensions were transferred to a disposable sizing cuvette to measure the size and placed in the device (Zetasizer nano-ZS, Malvern, UK). Three replicates were performed for each type of NPs and each assay consisted of 20 runs. The measurements were carried out at 25 °C.

To measure zeta potential of NPs, the samples were prepared in the same way. NP suspension was placed in dedicated cuvettes (DTS1070) and the surface zeta potential was measured three times (15 runs for each measurement, ZetaSizer Nano ZS, Malvern Instruments).

### 4.8. Scanning Electron Microscopy Observations

Nanoparticles of all the types (empty or with the addition of either GentAOT or GentS of 10%, 20%, or 30% of PLGA mass) were stuck to the metallic holder with carbon tape. Then, the 10 nm layer of carbon has been deposited on their surface to increase conductivity. The samples were then observed using a scanning electron microscope NOVA NANO SEM 200 at 5 mV and magnifications of 50,000 and 100,000.

### 4.9. In Vitro Drug Release

The drug release profiles were explored. To do so, each of the prepared hydrogels was immersed in a PBS buffer for 21 days. Briefly, 0.5 mL of each type of hydrogel was closed in dialysis bags, placed in vials filled with 20 mL of PBS, and constantly stirred at a speed of 100 rpm. The experiment was carried out at a temperature of 37 °C. To compare results, we also examined the release profiles of the drug from the NPs themselves. In brief, 2.22 mg of each type of NPs was suspended in 0.5 mL of water (the amount of NPs corresponding to the same volume of GG hydrogels) and placed in dialysis bags. The test was performed under the same conditions as those for hydrogels. In predetermined periods of time, 2 mL of PBS was collected and replaced with a fresh solution for up to 21 days. The amount of drug released was quantified using the OPA assay, as described previously.

### 4.10. Cytocompatibility

MC3T3 cells were cultured in Dulbecco’s Modified Eagle Medium (DMEM) supplemented with 10% FBS and 1% of antibiotics (penicillin-streptomycin mix) at 37 °C and 5% CO_2_. In their 2nd passage, they were seeded in 96-well TCPS cell culture plates (10,000 cells/well) and were incubated at 37 °C and 5% CO_2_ for 24 h. Meanwhile, GA30-NPs and GS30-NPs were sterilized by exposing them to UV light for 20 min. Then, they were collected and suspended in DMEM and diluted at several concentrations ranging from 0 to 1000 µg/mL. All concentrations of both types of suspensions were added to the wells (100 µL each) and incubated further in the same conditions for 72 h. After this time, the influence of suspension on cells was evaluated by metabolic AlamarBlue assay and live/dead fluorescent staining.

For AlamarBlue, resazurin was dissolved in PBS (0.11 mg/mL) and 10% *v*/*v* of this solution was added to DMEM supplemented as mentioned. The suspensions were removed, the residues of NPs were washed out with PBS, and 150 µL of the AlamarBlue reagent was added and incubated for 4 h. Then, 100 µL was collected from each well and transferred to the black 96-well plate. The fluorescence was measured at an excitation wavelength of 544 nm and an emission wavelength of 590 nm using a fluorometer (FluoStar OMEGA, BMG LabTech). The relative reduction of resazurin was calculated setting the range as 0%—fluorescence of the reagent incubated in empty wells, 100%—fluorescence of the reagent reduced in an autoclave. The values were compared to the control sample—cells cultured at a concentration of 0 µg/mL. For each sample, 3 wells were used.

The live/dead reagent was prepared by adding calcein AM and propidium iodide of 0.1% *v*/*v* each to PBS. The suspensions were removed from the wells and the residues of NPs were washed with PBS. 100 µL of live/dead reagent was added to each well and the plate was incubated in the darkness for 20 min. Then, the pictures were taken on a fluorescent microscope (ZEISS Axiovert 40 CFL) with a ZEISS HXP 120 C metal halide illuminator, using blue and green lights for live and dead cells, respectively. The pictures taken in the same spot were then combined.

### 4.11. Bactericidal Properties Evaluation

The antibacterial properties of the samples were evaluated using the Kirby–Bauer method (agar diffusion test). The suspension of the MRSA ATCC BAA 1681 strain was prepared in trypticasein soy broth (BioMaxima S.A., Lublin, Poland) at a concentration of 0.5 McFarland (1.5 × 10^8^ CFU/mL), and the bacteria were seeded on Mueller–Hinton agar. The NPs and hydrogel samples were sterilized by exposing them for 20 min to UV radiation. The holes in the agar of analogical sizes to the standard discs (i.e., 6 mm) in agar were cut out with a sterile pipette tip. Samples in the form of either PBS suspension (GA30-NPs and GS30-NPs in the concentration of 1000 µg/mL) or hydrogel (GG/GA30-NPs, GG/MIX-NPs, and GG/GS30-NPs) were placed in the holes in the amount of 100 µL of either NPs suspension or the hydrogels. The samples were then placed incubated for 4 h at 4 °C to allow for diffusion of the drugs. After that, the plates were moved to the incubator and kept at 37 °C for 24 h. Next, the diameters of the inhibition of the bacterial growth were measured. The experiment was carried out in triplicate for each sample group. Antimicrobial susceptibility test disc (6 mm in diameter, Oxoid, Hampshire, UK) soaked with sterile PBS was used as a negative control, as well as a GG/0-NPs prepared as described above. As a positive control, a standard gentamycin disc (10 µg dose) was used.

### 4.12. Statistics

The statistical analyses of the obtained data were done using a one-way analysis of variance (one-way ANOVA) followed by Tukey’s post hoc test. The analyses were performed using OriginLab software. The results are presented as mean ± standard deviation (SD) or standard error (SEM).

## 5. Conclusions

This study aimed to obtain the hydrophobic complex of gentamycin (GentAOT), which have a superior loading capacity in the hydrophobic polymers such as PLGA, to encapsulate it within PLGA NPs and compare its performance with the classic gentamycin sulfate (GentS) in the terms of physicochemical properties, loading capacity, release kinetics, as well as biological properties like cytocompatibility and antibacterial properties. NPs were tested alone or while suspended in an injectable hydrogel—one of the possible formulations for drug delivery to bone tissue.

The provided experiments showed that the drug loading capacity increases impressively with the use of GentAOT and has a superior release profile, especially during the first days of incubation in vitro. These features are improved without significant changes in the physicochemical properties. Only the cytotoxicity of the system increased while GentAOT was used. On the other hand, to deliver the same amount of drug in the case of GentS-loaded NPs, we would have to increase their concentration around 5 times. Therefore, we assume that it is possible to reduce the amount of the NPs required for the therapeutic effect, because of the use of GentAOT, and it should be possible to obtain the effect below the toxic concentration. Some features of the formulations might be adjusted by combining NPs loaded with hydrophobic and hydrophilic gentamycin.

Although GentAOT has already been tested several times in different formulations, it does not seem to go any further in the way toward clinical trials, despite its several advantages. In light of that, we encourage more extensive study in this area, especially in terms of GentAOT biocompatibility, to conclude the safe dose of it firmly, what is now lacking in the literature.

## Figures and Tables

**Table 1:** EE and DL of the batches obtained with either GentAOT or GentS.

Type of NPs	EE (%)	DL (%)
GA10-NPs	99.64 ± 0.09	10.79 ± 0.01
GA20-NPs	99.20 ± 0.02	17.73 ± 0.00
GA30-NPs	99.34 ± 0.01	22.54 ± 0.00
GS10-NPs	16.11 ± 2.78	1.45 ± 0.25
GS20-NPs	32.22 ± 1.82	5.33 ± 0.30
GS30-NPs	20.95 ± 5.15	4.87 ± 1.20

**Table 2:** Abbreviations of used NPs.

Acronym	The Drug Added to the NPs	Addition of Drug in mg per 100 mg PLGA
0-NPs	-	0
GA10-NPs	GentAOT	10
GA20-NPs	GentAOT	20
GA30-NPs	GentAOT	30
GS10-NPS	GentS	10
GS20-NPs	GentS	20
GS30-NPS	GentS	30

**Table 3:** Abbreviations of used PLGA nanoparticle-loaded hydrogel formulations.

Acronym	Amount Drug Added to 1 mL of GG	Amount NPs Added to 1 mL of GG
GG/0-NPs	-	4.44 mg 0-NPs
GG/GA30-NPs	1 mg GentS	4.44 mg GA30-NPs
GG/MIX-NPs	1 mg GentS	2.22 mg GA30-NPs & 2.22 mg GS30-NPs
GG/GS30-NPs	1 mg GentS	4.44 mg GS30-NPs

## Data Availability

Not applicable.
